# Discrepant diversity patterns and function of bacterial and fungal communities on an earthquake-prone mountain gradient in Northwest Sichuan, China

**DOI:** 10.3389/fmicb.2023.1217925

**Published:** 2023-08-22

**Authors:** Tianzhi Huang, Yingyan Wang, Xuemei Wang, Li Ma, Xueting Yang

**Affiliations:** ^1^Key Laboratory of Ecological Safety and Protection of Sichuan Province, Mianyang Normal University, Mianyang, China; ^2^College of Resources and Environmental Engineering, Mianyang Normal University, Mianyang, China; ^3^Research Center of Sichuan County Economy Development, Mianyang Normal University, Mianyang, China

**Keywords:** earthquake-prone areas, elevational gradient, enzyme activities, bacteria, fungi, microbial diversity

## Abstract

Patterns of microbial diversity on elevational gradients have been extensively studied, but little is known about those patterns during the restoration of earthquake-fractured alpine ecosystems. In this study, soil properties, soil enzyme activities, abundance and diversity of soil bacterial and fungal communities at four positions along a 2.6-km elevational gradient in the Snow Treasure Summit National Nature Reserve, located in Pingwu County, Southwest China. Although there were no significant changes in the soil chemical environment, bacterial and fungal communities were significantly different at different elevations. The overall fungal community presented an N-shaped diversity pattern with increasing elevation, while bacterial diversity decreased significantly with elevation. Changes in microbial diversity were associated with soil phosphorus, plant litter, and variations in dominant microbial taxa. Differences in enzyme activities among elevations were regulated by microbial communities, with changes in catalase and acid phosphatase activities mainly controlled by *Acidobacteria* and *Planctomycetaceae* bacteria, respectively (catalase: *p* < 0.001; acid phosphatase: *p* < 0.01), and those in β-glucosidase, sucrase, and urease activities mainly controlled by fungi. The β-glucosidase and sucrase were both positively correlated with *Herpotrichiellaceae*, and urease was positively correlated with *Sebacinaceae* (*p* < 0.05). These findings contribute to the conservation and management of mountain ecosystems in the face of changing environmental conditions. Further research can delve into the specific interactions between microbial communities, soil properties, and vegetation to gain deeper insights into the intricate ecological dynamics within earthquake-prone mountain ecosystems.

## 1. Introduction

Mountainous areas, which is far from human interference, encompass dramatic turnover in climate and biota over relatively short elevational distances and thereby provide powerful “natural experiments” to understand how biodiversity responds to environmental change. Based on above advantage, extensive studies on biodiversity of higher organisms (e.g., vascular plants, tree and birds) demonstrate different patterns of richness with increasing elevation (Guo et al., 2013; Dhyani et al., 2019). However, the effects of environmental variability along elevational gradients on taxonomic and functional diversity of soil bacteria and fungi are ambiguous, even though soil microbial communities are critical in regulating ecosystem functions and services (Ramirez et al., 2012; Liu et al., 2021). At present, the patterns of microbial diversity along elevational gradients include monotonic decreasing (Bryant et al., 2008), increasing (Margesin et al., 2009), hump-back (Han et al., 2018), and U-shape (Wang et al., 2012) patterns, but study also showed that soil microbial diversity did not vary with elevation (Singh et al., 2014). Soil enzyme activities mediated by microbe (e.g., s phosphatase, β-glucosidase, urease, sulphatase and dehydrogenase) play important role in carbon decomposition and nutrient recycling, and easily influenced by biotic and aerobic factors (Dasila et al., 2020). So, patterns of changes in microbial enzyme activities with increasing elevation are also inconsistent. In addition, trends in microbial diversity are not always consistent with those of microbial enzyme activity along elevational gradients (Ren et al., 2018). Because of their strong and inconsistent respond to environmental changes, microbial community and enzyme activities were regarded as optimum candidate to study elevational gradients.

At present, climate, soil properties, vegetation, and historical impacts are the four main determinants of composition and diversity of soil microbial communities along elevational gradients (Praeg et al., 2020). Climatic and edaphic variables (e.g., pH and C/N ratio) are frequently reported as key factors shaping elevational patterns of microbial communities (Donhauser and Frey, 2018), although such variables can have limited explanatory power. A primary reason for limited explanatory power is that microbial diversity may be controlled by processes operating at scales that do not match the temporal and spatial scales under study (Ladau and Eloe-Fadrosh, 2019). Particularly, because of small size and specific habitat requirements, soil microbes experience strongly buffered temperature and humidity extremes compared with those of open areas, with lower seasonal and interannual variability (De Frenne et al., 2019). In such buffered conditions, soil bacteria can survive in microrefugia despite unfavorable large-scale free-air conditions (Ashcroft, 2010). Therefore, soil micro-environment and historical impacts may be the main factors affecting soil microbial diversity and function over relatively short elevational distances. Earthquakes are one example of important historical impacts. The occurrence of it not only causes the disappearance or burial of the original vegetation layer, but also changes the environment and function of vegetation and soil continuously (Cheng et al., 2012). Therefore, the vegetation and soil environment of post-earthquake damaged mountain ecosystem is more complex than that of normal mountain ecosystem. However, the effects of earthquakes on soil microorganisms along elevational gradients are poorly understood.

The Snow Treasure Summit National Nature Reserve in the Longmen Mountain seismic zone in Northwest Sichuan, China, is a nature reserve of wild creatures that mainly protects giant pandas and their habitats. The Longmen Mountain seismic zone is one of the strongest seismic zones in Sichuan. Since 1169 AD, there have been 26 destructive earthquakes, 20 of which measured 6 or higher on the Richter scale. The 8.0 magnitude Wenchuan earthquake and the 7.0 magnitude Ya’-an earthquake occurred in the Longmen Mountain seismic zone. Years of effects of geological activities on the soils and vegetation in the Snow Treasure Summit National Nature Reserve have seriously affected the survival of wild creatures. In this study, soil bacterial and fungal communities were measured at four positions along a 1.0-km elevational gradient in the Snow Treasure Summit National Nature Reserve. The gradient was used to test the following hypotheses: (i) soil bacterial and fungal communities will have unique elevational patterns of diversity, but different environmental drivers will be correlated with those patterns; and (ii) differences in bacterial communities will be the dominant factor regulating microbial enzyme activity along the elevational gradient. Testing those hypotheses will generate information to help to maintain the sustainability of ecosystems in the Snow Treasure Summit National Nature Reserve and will also provide a relevant theoretical basis for the restoration and reconstruction of damaged soil ecosystems in the reserve.

## 2. Materials and methods

### 2.1. Study area

The study was conducted in the Snow Treasure Summit National Nature Reserve (31°59′31″–33°02′41″N, 103°50′31″–104°59′13″E), which is responsible for protecting the giant panda, golden monkey, and other rare wild animals and their habitats. The nature reserve, originally established in 1993, is in Sichuan Province, China, with a total area of 63,615 ha. Because the area is in the heart of the east slope of the Minshan Mountain system and has been protected by national and local governments for over 30 years, it is characterized by natural forest ecosystems with little human disturbance but disturbance from natural hazards. The study area is in the transition from subtropical mountain humid monsoon to cold climate zones in Northwest Sichuan. According to records of the Pingwu County meteorological station (3,250 m a.s.l.), mean annual maximum and minimum air temperatures are 37°C in July and −6.6°C in January, respectively. Mean annual precipitation is 1,300 mm. The dominant soil types are yellow and yellow-brown earths (Pu, 2020).

Forest coverage in the reserve is 85.0%, and evergreen broad-leaved forest is the typical zonal vegetation. *Taxus baccata* Linn (Taxaceae), *T. chinensis* (Taxaceae), *Davidia involucrata* Baill (Nyssaceae), and *Metasequoia glyptostroboides* Hu & W. C. Cheng (Taxodiaceae) are some rare tree species in the forest. Along the elevational gradient, the vegetation types range from evergreen broad-leaved forest (*<*2,000 m), to mixed coniferous broad-leaved forest (2,000–2,700 m), to coniferous forests (2,700–3,400 m), to alpine irrigation meadow (3,400–3,800 m), and to the vegetation of limestone beach and snow covers (>3,800 m) (Pu, 2020). The focus in this study was on the changes in evergreen broad-leaved and mixed coniferous broad-leaved forests, and thus, four elevations were selected: 1,600, 1,800, 2,200, and 2,600 m. A detailed description of site characteristics is provided in Supplementary Table S1.

### 2.2. Soil sampling

Soil samples were collected in July 2019. Four 20 m × 20 m permanent plots were systematically set up. Plots were roughly evenly distributed by elevation and collectively spanned 1026.19 m, from 1627.19 m to 2653.38 m a.s.l. Within each plot, six evenly distributed sampling points were set up, and six soil cores within each sampling point (10-cm depth directly below the litter layer, 5-cm diameter) were collected randomly and composited as a single sample. Visible plant roots and residues were removed before mixing. Soil samples were stored in plastic bags on ice. In the laboratory, samples were divided into two portions: one was air-dried and sieved through a 2-mm mesh screen for physicochemical analyses, and the other was stored at −80°C for DNA extraction and soil enzyme activity.

### 2.3. Chemical and enzyme activity analyses

Soil pH, soil organic carbon (SOC), total nitrogen (TN), total phosphorus (TP), total potassium (TK), available nitrogen (AN), available phosphorus (AP), and available potassium (AK) were measured using standard protocols following Liu et al. (2021). Detailed edaphic properties of the soil samples are provided in Table 1.

**Table 1:** Soil parameter information of the sampling sites along mountain gradient.

	pH	SOC (g. kg^−1^)	TN (g. kg^−1^)	TP (g. kg^−1^)	TK (g. kg^−1^)	AN (mg. kg^−1^)	AP (mg. kg^−1^)	AK (mg. kg^−1^)
1,600	8.16 ± 0.12a	9.37 ± 1.97c	1.08 ± 0.15c	0.64 ± 0.12ab	17.19 ± 1.36b	105.14 ± 24.69c	2.58 ± 0.21b	55.76 ± 12.25c
1,800	6.70 ± 0.10b	34.57 ± 8.26a	2.17 ± 0.51ab	0.79 ± 0.06a	15.64 ± 0.59b	233.90 ± 35.13b	4.70 ± 0.85a	58.81 ± 13.33c
2,200	5.33 ± 0.04d	22.83 ± 1.14ab	2.78 ± 0.14a	0.58 ± 0.00b	16.96 ± 0.41b	358.34 ± 13.04a	1.60 ± 0.16bc	131.87 ± 24.04a
2,600	5.68 ± 0.09c	14.08 ± 1.53c	1.72 ± 0.17bc	0.66 ± 0.01ab	23.43 ± 0.77a	228.27 ± 20.15b	1.14 ± 0.19c	83.47 ± 12.86c
*F*	190.253	6.479	6.133	1.843	16.342	17.672	12.177	4.624
*P*	0.00	0.003	0.004	0.172	0.00	0.00	0.00	0.013

Values (mean ± SE, *n* = 6) followed by different letters indicate statistically significant differences between sites (Tukey’s HSD test, *p* < 0.05). SOC, soil organic carbon; TN, total N; TP, total P; TK, total K; AN, available N; AP, available P; AK, available K; 1,600, 1,600 elevation; 1,800, 1,800 elevation; 2,200, 2,200 elevation; 2,600, 2,600 elevation.

Soil enzyme activities were measured as described by Guan (1986). Urease was determined by a colorimetric method using sodium phenol–sodium hypochlorite, and activity was expressed as mass of NH_4_^+^-N produced per unit time and per unit dry soil. Sucrase was determined by a 3,5-dinitrosalicylic acid colorimetric method, and activity was expressed in terms of glucose production per unit time and per unit dry soil mass. Catalase was determined by potassium permanganate titration, and activity was indicated by the volume of 0.02 mol/L KMnO_4_ consumed by 1 g of dry soil filtrate. β-glucosidase was determined by nitrophenol colorimetry, and activity was expressed by the content of p-nitrophenol produced per unit time and per unit dry soil. Activity of soil extracellular acid phosphatase (ACP) was estimated by measuring the release of *p*-nitrophenol from *p*-nitrophenyl phosphate (Tabatabai and Bremner, 1969). Potential ACP activities were expressed as μmol produced per g of soil (dry weight equivalent) within 1 h.

### 2.4. Preparation of 16S rRNA and its amplicon libraries and sequencing

The bacterial 16S rRNA gene was amplified using primers 338F/806R (Nan et al., 2016), and the fungal ITS gene was amplified using ITS1F/ITS2R (Bokulich and Mills, 2013) (ABI GeneAmp^®^ 9700, United States). The PCR was performed in a reaction mixture containing 12.5 μL of ABI Power SybrGreen qPCR Master Mix (Applied Biosystems), 0.5 μM each primer, 1 μL of 20 ng μL^−1^ template DNA, and sterile distilled water to make up a final volume of 25 μL. The qPCR program consisted of an initial denaturation at 95°C for 1 min and 30 cycles of 95°C for 15 s, 55°C for 30 s, and 72°C for 30 s. The PCR products were extracted from 2% agarose gel, further purified using an AxyPrep DNA Gel Extraction Kit (Axygen Biosciences, United States), and quantified using QuantiFluor^™^-ST (Promega, United States). Purified PCR products were paired-end sequenced (2 × 300 bp) on an Illumina MiSeq platform by Origingene Biotechnology Co., Ltd., Shanghai, China.

Raw sequence data were processed using the QIIME v1.9 pipeline, where sequences were quality filtered, chimera-checked, OTU-clustered, and taxonomically annotated (Caporaso et al., 2012). Operational taxonomic units (OTUs) were clustered with 97% similarity cutoff using UPARSE (v7.1). The taxonomy of each 16S rRNA and ITS gene sequence was analyzed using the SILVA r115 database (Quast et al., 2013) and the UNITE v5.0 database (Kõljalg et al., 2013), respectively. To eliminate the effects of different read numbers among the plots on the deduced compositions of bacterial and fungal communities, the number of sequences per soil sample was normalized to 35,100 reads for bacteria and 26,300 reads for fungi after removing the singletons. The each sample was rarefied to the identical number of 16S rRNA OTUs (3000) and ITS OTUs (600) for downstream analyses. The raw reads have been deposited in the National Center for Biotechnology Information (NCBI) Sequence Read Archive database (Accession Number: PRJNA913681).

### 2.5. Statistical analyses

Differences in soil properties and enzyme activities were tested using a random permutation test (Anderson and Walsh, 2013). Principal component analysis (PCA) was used to compare soil properties at different positions on the elevational gradient. Soil enzyme activities at different positions were compared using boxplots. Relative abundances of dominant taxa of soil bacteria and fungi in each sample were calculated and ranked. The calculation of relative abundance was based on the proportional frequencies of the DNA sequences from all samples that could be classified at the phylum level.

An OTU table was used for downstream alpha and beta analyses. Taxonomic alpha diversity of soil bacteria and fungi was estimated using OTU richness, Pielou’s evenness, and the Shannon–Wiener diversity index. To calculate taxonomic beta diversity, the Bray–Curtis index for abundance-weighted dissimilarity was used. Phylogenetic beta diversity was calculated using both weighted and unweighted UniFrac distance. To examine the elevational differences in compositional dissimilarities, principal coordinates analysis (PCoA) and permutational multivariate analysis of variance (PERMANOVA) were generated using “vegan” package in the R statistical software platform (v2.15.0). Differences in taxa among elevations were analyzed using linear discriminant analysis (LDA) effect size (LEfSe) analysis^1^ (Segata et al., 2011) and were visualized using the R package DESeq2 (Love et al., 2014). Enriched genera (up) and depleted genera (down) were defined as genera with differences in relative abundances (*p* < 0.1) between two contiguous sites along the elevational gradient. Partial Mantel tests were used to test the correlations between environmental variables and bacterial and fungal taxa.

For the predicted functional groups, a linear model of Spearman’s rank correlations was used to examine the relations between enzyme activities and relative abundances of bacterial and fungal groups, with relations visualized using the Cytoscape package v3.2.1 (Shannon et al., 2003).

## 3. Results

### 3.1. Soil properties and enzyme activities on the elevational gradient

**Figure 1:**
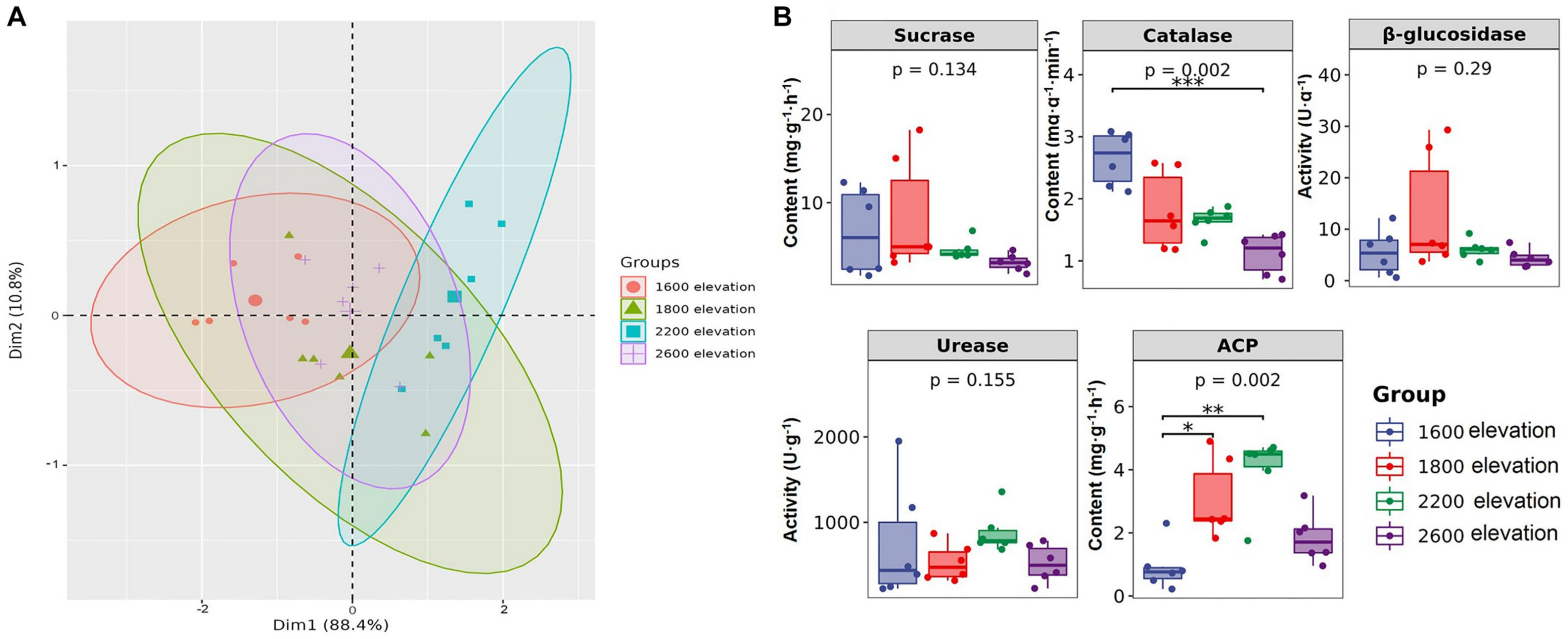
**(A)** Principal component analysis of soil properties, and **(B)** differences in soil enzyme activity along an elevational gradient in the Snow Treasure Summit National Nature Reserve. Difference significance: *p < 0.05; **p < 0.01; ***p < 0.001.

Soil properties were significantly different along the gradient, except for total P (Table 1). Soil pH was the lowest at 2,200 m, but the contents of TN, AN, and AK were the highest. Contents of TP, AP, and SOC were the highest at 1,800 m. Although individual soil properties were significantly different among sites on the elevational gradient, overall soil environments were not clearly separated by elevation along the first principal component of the PCA, which explained over 88% of the variation in soil properties. Thus, distinct soil environments did not develop on the mountain gradient in Snow Treasure Summit National Nature Reserve (Figure 1A). Among enzyme activities (Figure 1B), sucrase and catalase activities decreased along the elevational gradient. Activities of urease and ACP were the highest at 2,200 m. The activity of β-glucosidase was slightly higher at 1,800 m than at other elevations. The changes in activities of β-glucosidase, urease, and sucrase along the gradient were not significant. In addition, catalase was negatively correlated with TK and positively correlated with pH (*p* < 0.05; Supplementary Table S2). Activity of β-glucosidase was positively correlated with SOC, TP, and AP (*p* < 0.05), and activity of ACP was negatively correlated with pH and positively correlated with SOC, TN, AP, and AK (*p* < 0.05; Supplementary Table S2).

### 3.2. Contrasting patterns with increasing elevation: N-shaped for fungal diversity, decreasing for bacterial diversity

**Figure 2:**
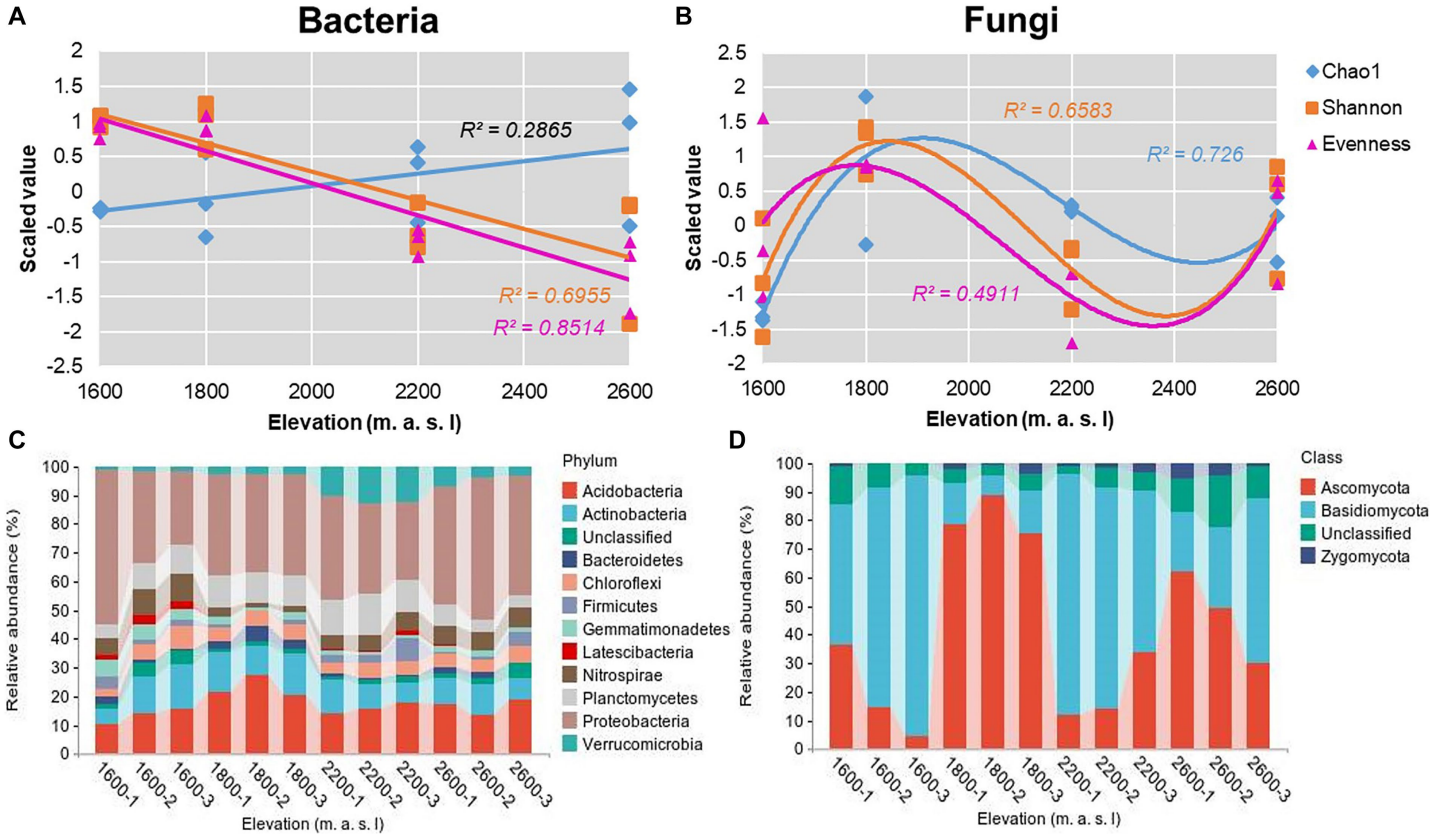
Elevational patterns for α diversity indices of overall **(A)** bacterial and **(B)** fungal communities. Scaled values were for z-transformed Chao1, evenness, and Shannon indices. Trends across elevations were determined using Gaussian process regression. **(C)** Twelve most abundant phyla in bacterial communities and **(D)** most abundant phyla in fungal communities along an elevational gradient in the Snow Treasure Summit National Nature Reserve.

The overall fungal community showed significant N-shaped patterns for richness, evenness, and Shannon diversity indices (Figure 2B) along the elevational gradient, whereas evenness and diversity indices of the overall bacterial community decreased significantly with elevation (Figure 2A). The Chao1 index of bacteria ranged from 3,707 to 6,153 (Supplementary Table S3), and the Shannon diversity index varied from 5.69 to 6.89 (Supplementary Table S3). Notably, the Chao1 index of bacteria increased with elevation, whereas the Shannon diversity index decreased. The lowest Shannon indices of bacteria were detected at 2,600 m, but the lowest Chao1 indices were detected at 1,600 m (Figure 2A). According to Pearson tests, the Shannon index of soil bacteria was positively correlated with AP (*p* = 0.024) and negatively correlated with TK and AK (*P*_TK_ = 0.017 and *P*_AK_ = 0.037; Supplementary Table S4). For bacteria, the phyla Proteobacteria, Acidobacteria, and Actinobacteria collectively accounted for 50.6–71.9% of the total sequences along the elevational gradient (Figure 2C). The trends in relative abundances of bacterial phyla were not similar to those in α diversity (Supplementary Figure S1A), but some bacterial phyla were correlated with soil properties (Supplementary Table S5). In addition, the dominant genera were *Massilia*, *Bradyrhizobium*, *Nitrosomonadaceae*, and *Xanthobacteraceae* in *Proteobacteria*; *Acidobacteria* and *Candidatus Solibacter* in *Acidobacteria*, and *Pseudarthrobacter*, *Gaiellales*, and *Acidimicrobiales* in *Actinobacteria* (Supplementary Figure S2).

The Chao 1 index of fungi ranged from 115 to 1,068, and the Shannon diversity index varied from 2.13 to 4.64 (Supplementary Table S3). Overall, the trends in fungal Chao1 and Shannon diversity indices were similar, with values increasing from 1,600 to 1,800 m, then declining at 2,200 m, followed by significant increases from 2,200 to 2,600 m. In Pearson tests, the Shannon index of soil fungi was positively correlated with TP (*p* = 0.016), and the Chao1 index was positively correlated with SOC (*p* = 0.001; Supplementary Table S4). In fungal communities, the phyla Ascomycota and Basidiomycota collectively accounted for 78.9–95.2% of the total sequences along the elevational gradient (Figure 2D). The trend in relative abundance of Ascomycota was similar to that of α-diversity (Supplementary Figure S1). *Ascomycota* and *Leotiomycetes* were the dominant genera in Ascomycota, and *Agaricomycetes* was dominant in Basidiomycota (Supplementary Figure S2).

### 3.3. Effect of elevation on compositional dissimilarities of bacterial and fungal communities

**Figure 3:**
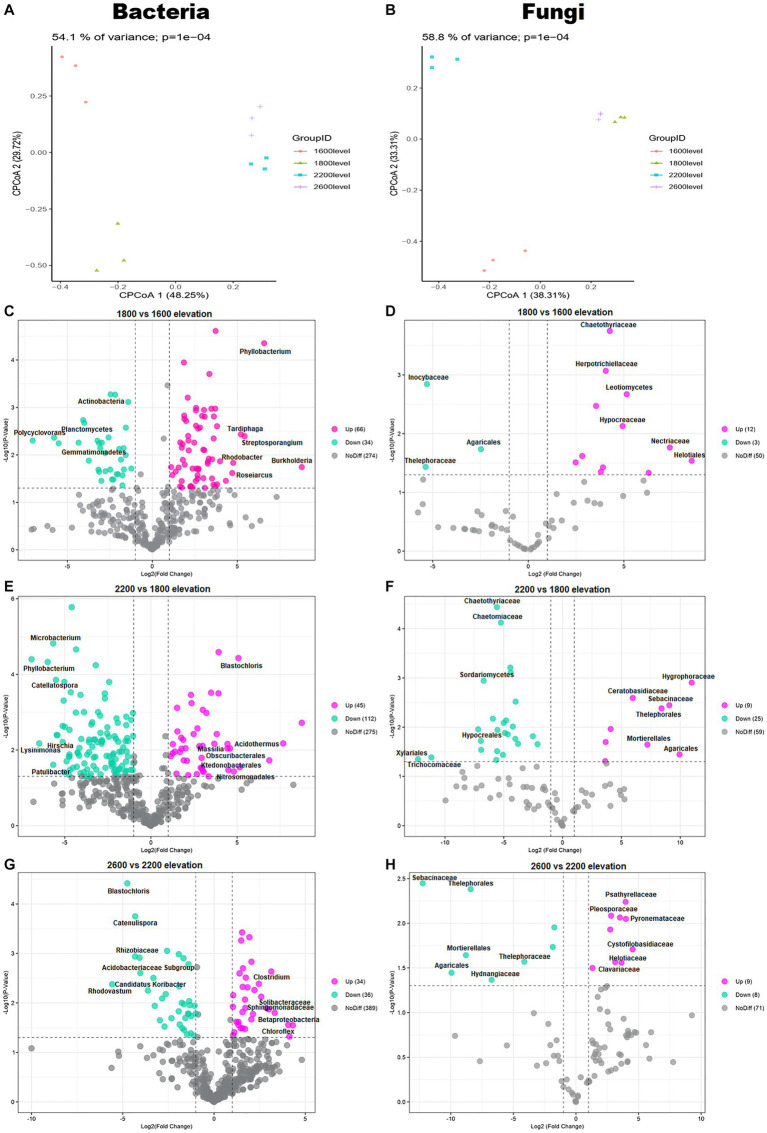
Principal coordinates analysis (PCoA) plots of trends in the composition of **(A)** bacterial and **(B)** fungal communities based on Bray–Curtis dissimilarity. Volcano plots illustrating taxa of **(C,E,G)** bacteria and **(D,F,H)** fungi with significant differences among elevations in the Snow Treasure Summit National Nature Reserve. Each point represents an individual taxon.

The composition of bacterial and fungal communities differed with elevation, as shown in PCoA plots based on Bray–Curtis distance (Figures 3A,B). According to PERMANOVA, the compositional dissimilarities among elevations were significant (*p* < 0.001; Figures 3A,B). The dissimilarity of bacterial and fungal communities significantly and unimodally increased with increasing elevation distance (Figures 3C–H). Compared with communities at 1,600 m, at 1,800 m, 66 genera of bacteria and 12 genera of fungi were enriched. Compared with 1,800 m, at 2,200 m, 112 genera of bacteria and 25 genera of fungi were depleted. Therefore, whether bacteria or fungi, the greatest differences were at 1,800 m. The bacterial genus *Phyllobacterium* increased significantly at 1,800 m (Figure 3C). The fungal families *Chaetothyriaceae* and *Hypocreaceae* also increased significantly at 1,800 m (Figure 3D). In addition, the family *Hypocreaceae* increased significantly with elevation, except at 2,600 m (Figures 3D,F,H). The number of genera with differences was similar at 2,200 and 2,600 m, but the differences were not same.

**Figure 4:**
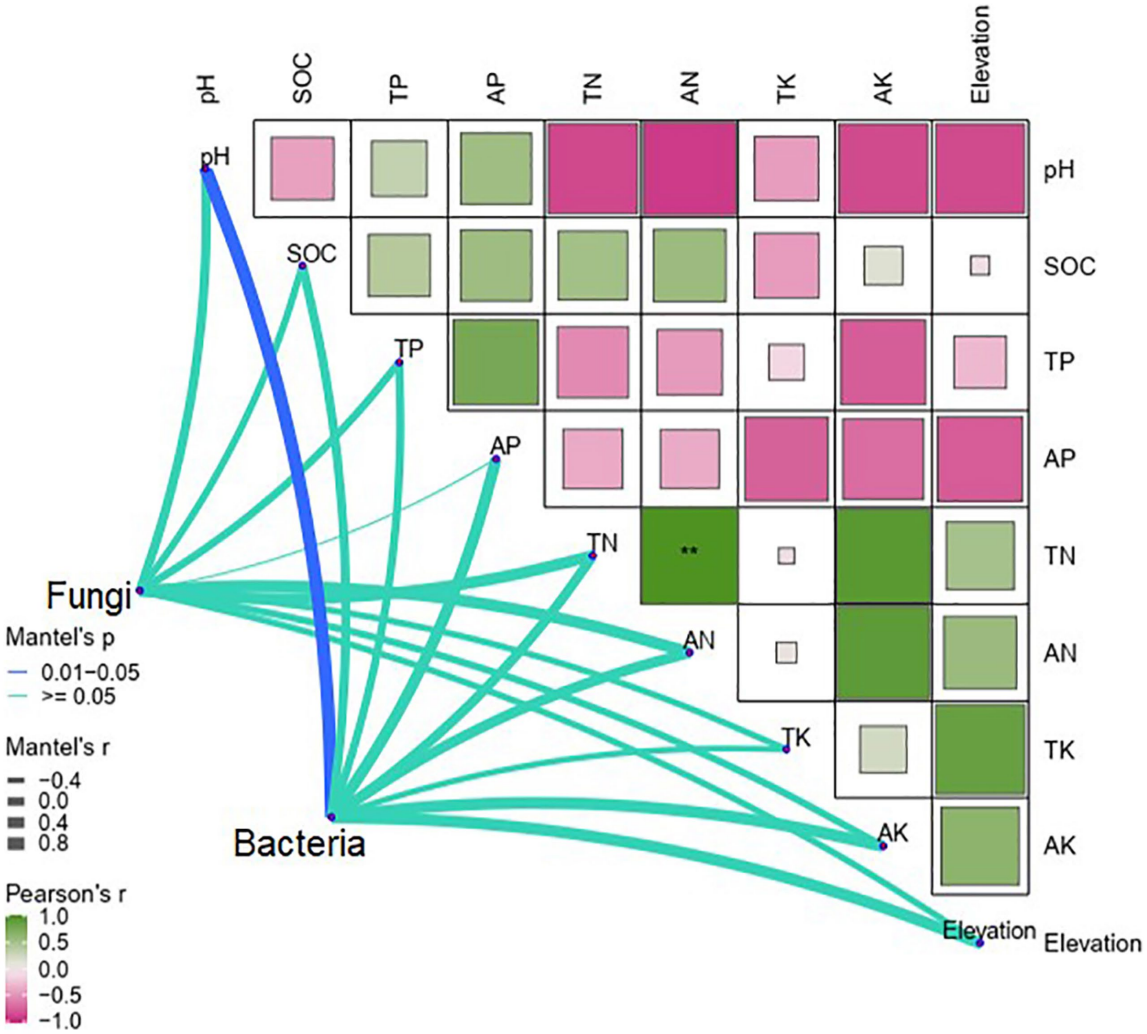
Mantel tests of differences in soil bacteria and fungi with soil properties along the elevational gradient in the Snow Treasure Summit National Nature Reserve and Pearson tests of correlations between soil bacteria and fungi and soil properties along the elevational gradient. TP, total phosphorus; AP, available phosphorus; TN, total nitrogen; AN, available nitrogen; TK, total potassium; AK, available potassium; pH, soil pH; SOC, soil organic carbon. Correlation significance: **p < 0.01.

Pearson correlations indicated that TK (*p* < 0.01) and AK (*p* < 0.05) were positively correlated with elevation, whereas pH and AP were negatively correlated with elevation (*p* < 0.001; Figure 4). The Mantel test revealed that soil pH was the only determinant of differences in soil bacteria between elevations (*p* < 0.05). By contrast, differences in soil fungi were not significantly affected by elevation and soil properties (Figure 4).

### 3.4. Predicted functional groups of bacteria and fungus

**Figure 5:**
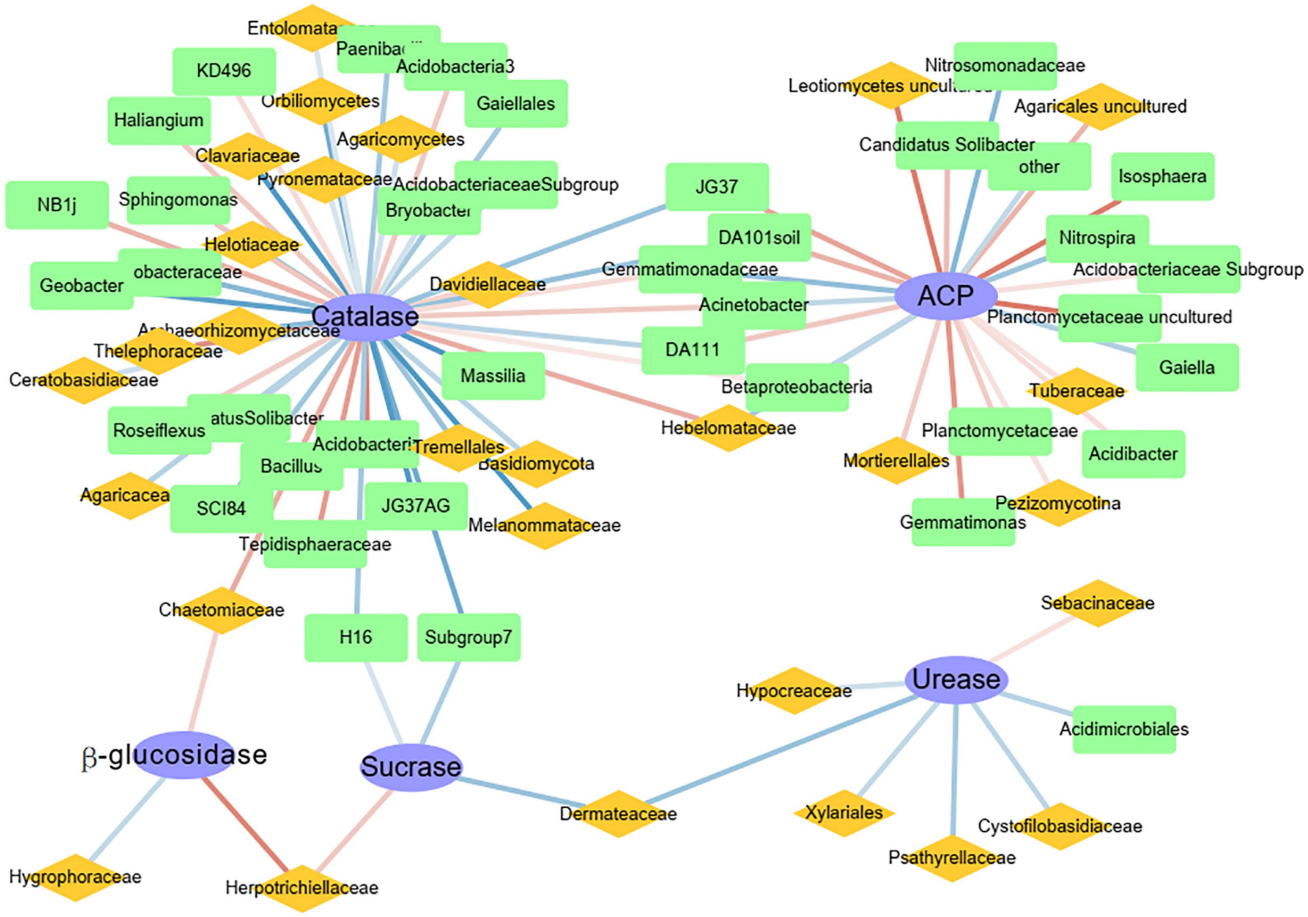
Spearman network analysis of the correlations between the 50 most abundant genera of bacteria (green squares) and fungi (yellow diamonds) and enzyme activities (blue ellipses). Blue lines, negative correlations; red lines, positive correlations. The darker the line, the greater the R-value.

Network analysis was used to determine the correlations between the top 50 genera of bacteria and fungi and activities of different enzymes (Figure 5). The network analysis showed that each enzyme had potential functional genera of bacteria and fungi. The highest number of functional genera was associated with catalase, whereas the lowest number of functional genera was associated with β-glucosidase, and all genera were fungal. Catalase was significantly positively correlated with the bacteria *Acidobacteria* and *Tepidisphaeraceae* and the fungi *Thelephoraceae* and *Chaetomiaceae* (*p* < 0.001). The enzyme ACP was significantly positively correlated with the bacteria *Planctomycetaceae* and *Isosphaera* and the fungi *Agaricales* and *Leotiomycetes* (*p* < 0.01). Urease was significantly positively correlated with only the fungal family *Sebacinaceae* (*p* < 0.05). In addition, sucrase and β-glucosidase were both positively correlated with the fungal family *Herpotrichiellaceae*. Although catalase and ACP shared some functional genera, the functional genera did not have the same effects. For example, *Gemmatimonadaceae*, a family of bacteria, was significantly positively correlated with catalase and negatively correlated with ACP.

## 4. Discussion

### 4.1. Differences in soil properties along the elevational gradient

The change in SOC along the elevational gradient in the Snow Treasure Summit National Nature Reserve was not consistent with increases in SOC with elevation in previous study (Zhang et al., 2021), which might be because of the type of the litter and other inputs into soil (Zhang et al., 2022). Compared with vegetation at other elevations, at 1,800 m, the more complex shrub community and hardwood species increased the thickness of the litter layer (Li et al., 2021) (Supplementary Table S1), which led to an increase in SOC. Additionally, the increase in SOC might be the main factor driving increases in TP and AP at 1,800 m, because organic matter formed during litter decomposition tends to fix soil P (Fekete et al., 2014). Simultaneously, AP is a by-product in the process of further microbial metabolism of organic matter that accumulates continuously (Luo et al., 2017). Many environmental conditions in montane ecosystems often covary with elevation (Looby and Martin, 2020). However, in this study, the soil environment at different elevations was not significantly different. It was hypothesized that the absence of differences might be associated with local, frequent seismic secondary disasters. After each secondary disaster, large areas of soil are disrupted and migrate (Cheng et al., 2012), which eliminates differences in soil environments at different elevations.

### 4.2. Soil conditions co-mediate differences in microbial communities on the elevation gradient

Along the elevational gradient in the Snow Treasure Summit National Nature Reserve, fungal communities showed N-shaped changes in diversity indices. By contrast, in bacterial communities, the richness index increased with elevation and evenness and Shannon indices decreased. The different responses of soil bacterial and fungal community diversity are consistent with results in study (Shen et al., 2020), suggesting different responses of microbial groups to environmental variations along elevation gradients. Although a global study on topsoil microorganisms demonstrated that niche differentiation between fungi and bacteria was associated with contrasting responses of diversity to soil pH (Bahram et al., 2018), in this study, the contrasting responses of fungal and bacterial diversity were associated with soil P or SOC (Supplementary Table S4). Soil texture is a key factor and can lead to an important role for P in niche differentiation between fungi and bacteria. Because the topsoil of both mountain brown soil and mountain yellow-brown soil has high levels of humus, organic P forms easily in soil (Nannipieri et al., 2011), and thus, P becomes an important factor limiting the growth and development of bacteria and fungi (Yang et al., 2022). Bacteria and fungi also have important differences in how they obtain P. Bacteria secrete alkaline or acid phosphatase to obtain P (Romanyà et al., 2017), whereas fungi obtain P by symbiosis with plants, with roots secreting acid phosphatase (Shenoy and Kalagudi, 2005). Therefore, temperature may be the main factor affecting bacterial P metabolism (Bahram et al., 2018), whereas vegetation may be the main factor affecting fungal P metabolism (Karandashov and Bucher, 2005), and the difference could lead niche differentiation between fungi and bacteria.

In this work, diversity of soil bacteria decreased with the increase in elevation, similar to results in previous study (Zhang et al., 2022). However, differences in relative abundances of bacterial phyla with elevation might not only be attributed to soil moisture and temperature (Ren et al., 2018) but also to other soil properties (Supplementary Table S5). The U-shaped relative abundance of *Nitrospirae* with increasing elevation (Supplementary Figure S1A) might be associated with its preference for C-limited soil conditions (Feng et al., 2017) and therefore the hump-shaped soil total C content with increasing elevation (Table 1). In addition, relative abundances of *Verrucomicrobia* and *Gemmatimonadetes* were both correlated with soil pH and N, but the correlations were the opposite. According to Xu et al. (2020), the *Verrucomicrobia* are considered an oligotrophic group, but moderate increases in N can boost its abundance at low soil pH (Ramirez et al., 2012). The ratio between abundances of *Proteobacteria* and *Acidobacteria* (P/A) can indicate soil nutrient status, with higher values indicating more nutrient-rich soils. Therefore, changes in relative abundances of bacterial phyla with elevation may be attributed to differences in ecological strategies (Ivanova et al., 2016). Although decreases in diversity of soil bacteria with elevation are well known, increases in the Shannon–Wiener index of diversity of soil fungi with elevation are rarely reported. Theoretically, for microbial populations at high elevations, inhabitable environments due to desiccation and severely nutrient-limited soils at low temperatures can pose extreme stresses, which lead to reductions in activities and diversity (Donhauser and Frey, 2018). However, Miyamoto et al. (2014) reported that fungal diversity showed a hump-shaped pattern with increasing elevation from 1,100 to 2,250 m on Mount Fuji, which is similar to the pattern in this work (Figure 2B). Therefore, there may be environmental factors affecting fungal diversity other than desiccation and low temperatures. In further analysis, relative abundance of the dominant fungal phylum Ascomycota showed an N-shaped response to increasing elevation (Figure 2D), which contributed to the overall fungal community pattern (Figure 2B). The response was generally consistent with the changes in SOC and P resulting from abundant vegetation. By contrast, relative abundance of the second most dominant phylum, Basidiomycota (which contains abundant mycorrhizal fungal taxa), was inversely correlated with those changes. The response of Basidiomycota might be because plants rely on the mycorrhizal fungus symbiotic exchange system for nutrient uptake. However, when levels of soil nutrients, such as N and P, are adequate, plants are less dependent on mycorrhizal fungi and reduce the quantity of C flowing into the rhizosphere, thus initiating a decrease in mycorrhizal fungal taxa (Eisenlord and Zak, 2010). Therefore, the most important influence of vegetation on fungal diversity may be determining the quantity and quality of litter substrates.

### 4.3. Dissimilarities of microbial communities respond to different ecological drivers

In general, the dissimilarities in both bacterial and fungal communities unimodally increased with elevation, but the increases were influenced by different ecological drivers. Many studies show that soil pH and soil organic matter have significant effects on soil microbial communities (Wang et al., 2015; Shen et al., 2019). The results in this study are consistent with that conclusion, and soil pH significantly influenced the relative abundance of bacteria between elevations (Figure 4). Generally, bacteria have a relatively narrow pH tolerance range for growth. However, the sites on the gradient in this study had a wide range of soil pH values, from 5.33 to 8.16 (Table 1), resulting in a strong correlation between bacteria and soil pH. A similar correlation is observed in other elevational studies (Wang et al., 2015; Shen et al., 2019). Additionally, soil pH was highly correlated with the relative abundance of two dominant bacterial phyla, *Verrucomicrobia* and *Gemmatimonadetes*. According to difference analysis, the bacterial genus *Phyllobacterium* increased significantly at 1,800 m, which was associated with increased richness in plant coverage. The association was likely because *Phyllobacterium* transiently or continuously inhabits plant leaves, and its numbers vary among plant species (Coutinho and Bophela, 2021). Thus, the thick litter layer provides an excellent environment for the genus. Although there was dissimilarity in fungal communities among elevations, the dissimilarities were not controlled by single soil property. Fungi have important ecological roles as decomposers, mutualists, and pathogens of plants (Shen et al., 2020). Thus, because of the many differences in fungi, such as *Chaetothyriaceae* and *Hypocreaceae*, that occurred at 1,800 m where the vegetation was most diverse, it was hypothesized that there was a significant difference in the plant type-fungal community along the elevational gradient in this study. Members of *Chaetothyriaceae* have been reported since the 19th century as sooty molds, which gain nutrients from sugary exudates, after their ascomata were found adpressed to the surface of leaves and stems (Tian et al., 2021). Culture and sequence data are available for only a fraction of the *Chaetothyriaceae*. Appropriate description of the *Chaetothyriaceae* is therefore not yet possible. However, life cycles of the family have been described in relation to plants, especially lichens, which led to the expansion of cytochromes, providing windows of opportunity for diversification (Quan et al., 2020). Similarly, most *Hypocreaceae* are free-living, avirulent plant symbionts, commonly in all types of soils inhabiting root ecosystems (Barman et al., 2021). The fungi can protect plants by improving vegetative growth and stimulating natural resistance (Barman et al., 2021). Therefore, both *Chaetothyriaceae* and *Hypocreaceae* were strongly influenced by vegetation type and litter. However, the type of vegetation involved is uncertain, and that determination will be part of future research.

### 4.4. Linkages between soil microbial communities and enzyme activity

In this study, sucrase and catalase activities decreased with elevation, but those of urease, ACP, and β-glucosidase did not (Figure 1B). It was hypothesized that in addition to soil chemical factors, soil microbiological factors also had important effects on enzyme activities (Salazar et al., 2011; Jain and Pandey, 2016; Looby and Treseder, 2018). Each soil enzyme is regulated by different soil nutrients (Salazar et al., 2011; Jain and Pandey, 2016), which is consistent with the results in this study. Soil chemical factors associated with catalase were consistent with those affecting bacterial community diversity (Supplementary Tables S3, S5), that is, catalase activity was regulated by the indirect influence of the soil environment on bacterial communities. In addition, the correlation between catalase and dominant microbes (Figure 5) also supported that conclusion, because catalase was almost entirely regulated by bacteria. In previous studies characterizing humus intensity and organic matter conversion rate in soil, catalase has indeed been shown to have a close relation with bacteria (Zhu et al., 2018). Notably, *Acidobacteria* and *Tepidisphaeraceae* were significantly positively correlated with catalase (Figure 5). In a recent report, *Acidobacteria* were responsible for a substantial fraction of catalase transcripts to increase H_2_O_2_ detoxification in a western Lake Erie *Microcystis* bloom community, despite relatively low abundance (Smith et al., 2022). Therefore, combined with results in this study, *Acidobacteria* express catalase well in both terrestrial and aquatic ecosystems. *Thelephoraceae* is a new family in the phylum *Planctomycetes*, and therefore, sufficient reports about its correlation with catalase are not yet available.

In contrast to catalase, ACP did not decrease with elevation but showed a hump-shaped pattern (Figure 1), which was possibly related to its complex sources. First of all, plant roots, microorganisms, and fauna all secrete ACP to increase soil P availability (Spohn and Kuzyakov, 2013). However, soil ACP was mainly regulated by bacteria in this study (Figure 5). Because bacteria are most likely to secrete ACP in neutral or acidic soil environments (Fraser et al., 2017), which could be why acid phosphatase was negatively correlated with soil pH value (Supplementary Table S2). In addition, bacteria secrete ACP as a by-product of C metabolism (Luo et al., 2017). So, ACP was significantly positively correlated with organic matter (Supplementary Table S2). Although fungi in the form of mycorrhizae promote the secretion of ACP by plant roots (Eisenlord and Zak, 2010), the highest content of ACP was not at 1,800 m, which had the highest vegetation richness, indicating that fungi were not the dominant source of ACP in this region. The *Planctomycetaceae* includes major bacteria in global N and C cycles (Strous et al., 1999). In addition, the *Planctomycetaceae* are also typical phosphate-solubilizing bacteria, which contain *phoD* or *phoC* genes (Zhao et al., 2022). Therefore, the *Planctomycetaceae* have important roles in biogeochemical cycles.

Except for ACP and catalase, the other enzymes were most influenced by fungi. The enzyme β-glucosidase was only controlled by fungi, such as *Hygroraceae*, *Herpotrichiellaceae*, and *Chaetomiaceae* (Figure 5), which might have been influenced by plant litter. Because β-glucosidase activity was correlated with SOC, TP, and AP (Supplementary Table S2), those fungi were controlled by those nutrients. As noted in 4.1, there were relations between plant litter and SOC and P. *Herpotrichiellaceae* were important fungi in this ecosystem, because the family was positively correlated with β-glucosidase and sucrase (Figure 5). However, the *Herpotrichiellaceae* includes well known pathogens, with several species associated with infections in humans and animals (Yang et al., 2021). In fact, according to metagenomic data analysis, the largest number of species in the *Herpotrichiellaceae* are found in soil-associated material and in plants, because the oligotrophic nature of these fungi enables them to survive in adverse environments where common saprobes are absent (Costa et al., 2020). Unfortunately, research on the family remains in its infancy, and little is known about its various metabolic capabilities. Urease is responsible for decomposition of N compounds. In this study, urease was not correlated with soil properties (Supplementary Table S2) but was significantly positively correlated with *Sebacinaceae* (Figure 5). Molecular and ultrastructural studies reveal a broad diversity of mycorrhizal associations involving members of the heterobasidiomycetous *Sebacinaceae*, which are fungi, owing to the inconspicuous basidiomes, have been often overlooked. As mycobionts, the fungi in the family have important roles in N accumulation in soils (Yang et al., 2022). Fungi in the *Sebacinaceae* are enriched at the early climax forest stage and decrease soil organic N accumulation by expediting the decomposition of soil organic matter. As a result, the *Sebacinaceae* use C to convert organic N for use by plant or microbes. Thus, a significant positive correlation between *Sebacinaceae* and urease is plausible. To conclude, the significant differences in soil enzyme activities among elevations were caused by differences in microbial communities.

## 5. Conclusion

In this study, although the soil chemical environment was not significantly affected by elevation, bacterial and fungal communities were significantly different at different elevations. Diversity of overall fungal community showed a significant N-shaped pattern with an increase in elevation, whereas diversity of the overall bacterial community decreased significantly with an increase in elevation. Differences were correlated not only with soil P and plant litter but also with changes in dominant microbes. The taxa of bacteria and fungi with differences were the richest at 1,800 m, but only bacteria with differences were significantly correlated with soil pH. Soil enzyme activities were different among elevations and were controlled by different microbes. The enzymes catalase and ACP were mainly controlled by *Acidobacteria* and *Planctomycetaceae* bacteria, respectively, whereas β-glucosidase, sucrase, and urease were mainly controlled by fungi. β-glucosidase and sucrase were both positively correlated with *Herpotrichiellaceae*, and urease was positively correlated with *Sebacinaceae*. Thus, the changes in microbial diversity and soil enzyme activities along the elevational gradient were associated with changes in the soil chemical environment and plant litter.

## Data availability statement

The datasets presented in this study can be found in online repositories. The names of the repository/repositories and accession number(s) can be found in the article/Supplementary material.

## Author contributions

TH proposed the study, designed the experiments, and participated in supervision. YW analyzed data and wrote the manuscript. XW and LM conducted experiments. XW and XY participated in field investigation. All authors contributed to the article and approved the submitted version.

## Funding

This work was supported by the National Natural Science Foundation of China (No. 32001232 and 32101363) and Open Fund Project of Key Laboratory of Ecological Safety and Protection of Sichuan Province (No. ESP1701).

## Conflict of interest

The authors declare that the research was conducted in the absence of any commercial or financial relationships that could be construed as a potential conflict of interest.

## Publisher’s note

All claims expressed in this article are solely those of the authors and do not necessarily represent those of their affiliated organizations, or those of the publisher, the editors and the reviewers. Any product that may be evaluated in this article, or claim that may be made by its manufacturer, is not guaranteed or endorsed by the publisher.

## Footnotes

^1^http://huttenhower.sph.harvard.edu/galaxy/
