# Supplementary material for: Discrepant diversity patterns and function of bacterial and fungal communities on an earthquake-prone mountain gradient in Northwest Sichuan, China
Source: Front Microbiol. 2023 Aug 22;14:1217925. doi: 10.3389/fmicb.2023.1217925 (PMC10477999; doi:10.3389/fmicb.2023.1217925)
Supplement: Supplementary file 1 [file Data_Sheet_1.docx]

Supplementary Material

Discrepant diversity patterns and function of bacterial and fungal communities on an earthquake-prone mountain gradient in Northwest Sichuan, China

Tianzhi Huang*, Yingyan Wang, Xuemei Wang, Li Ma, and Xueting Yang

*** Correspondence:** Tianzhi Huang: [huangtianzhi1@gmail.com](mailto:huangtianzhi1@gmail.com)

# Supplementary Figures and Tables

## Supplementary Figures


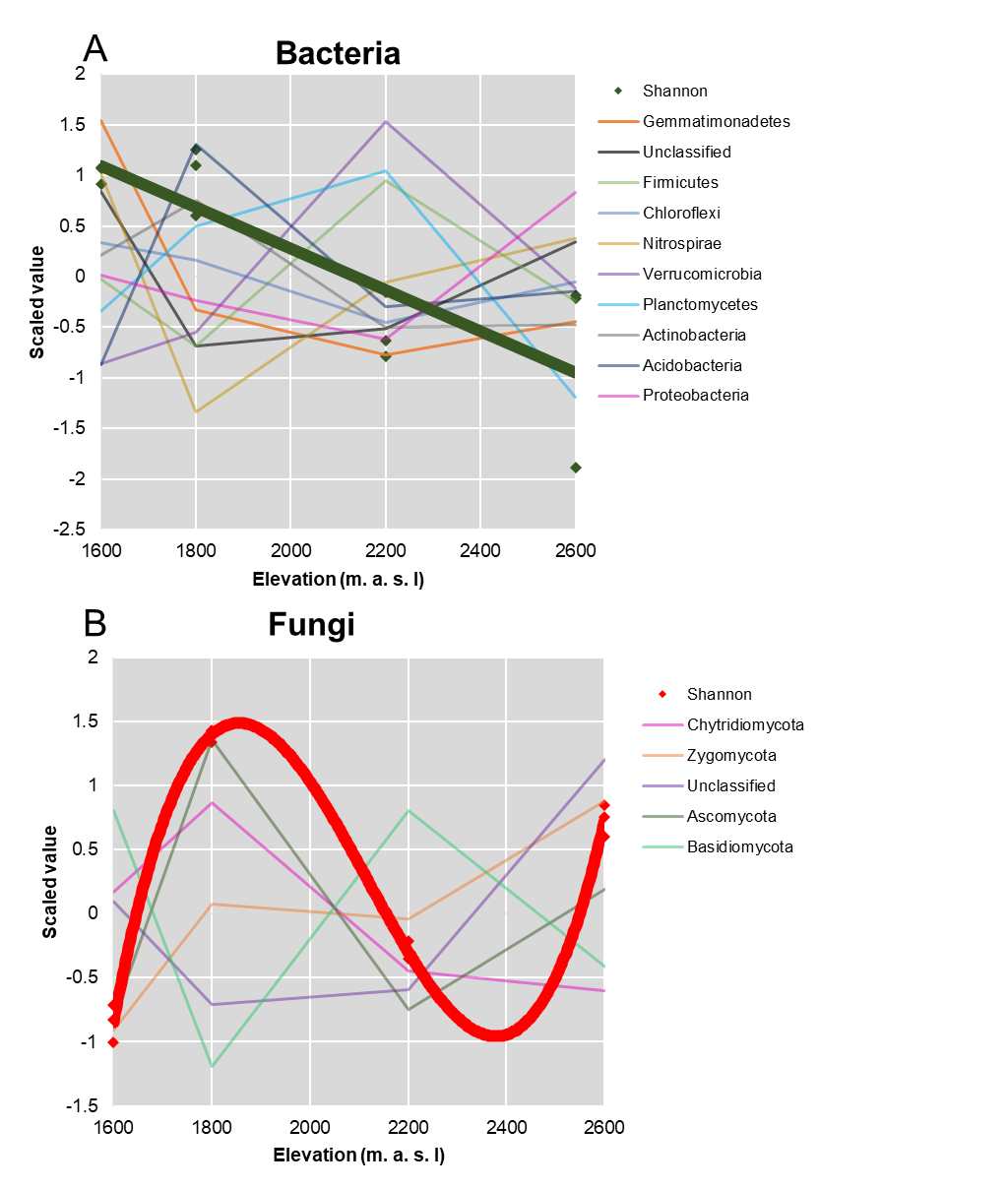


**Figure. S1** Elevational patterns for the Shannon diversity of bacterial whole community (with green points and green fitting curve) and the relative abundance of dominant phyla (A). Elevational patterns for the Shannon diversity of bacterial whole community (with red points and red fitting curve) and the relative abundance of dominant phyla (B). Scaled value, z-transformed Shannon index and relative abundance.


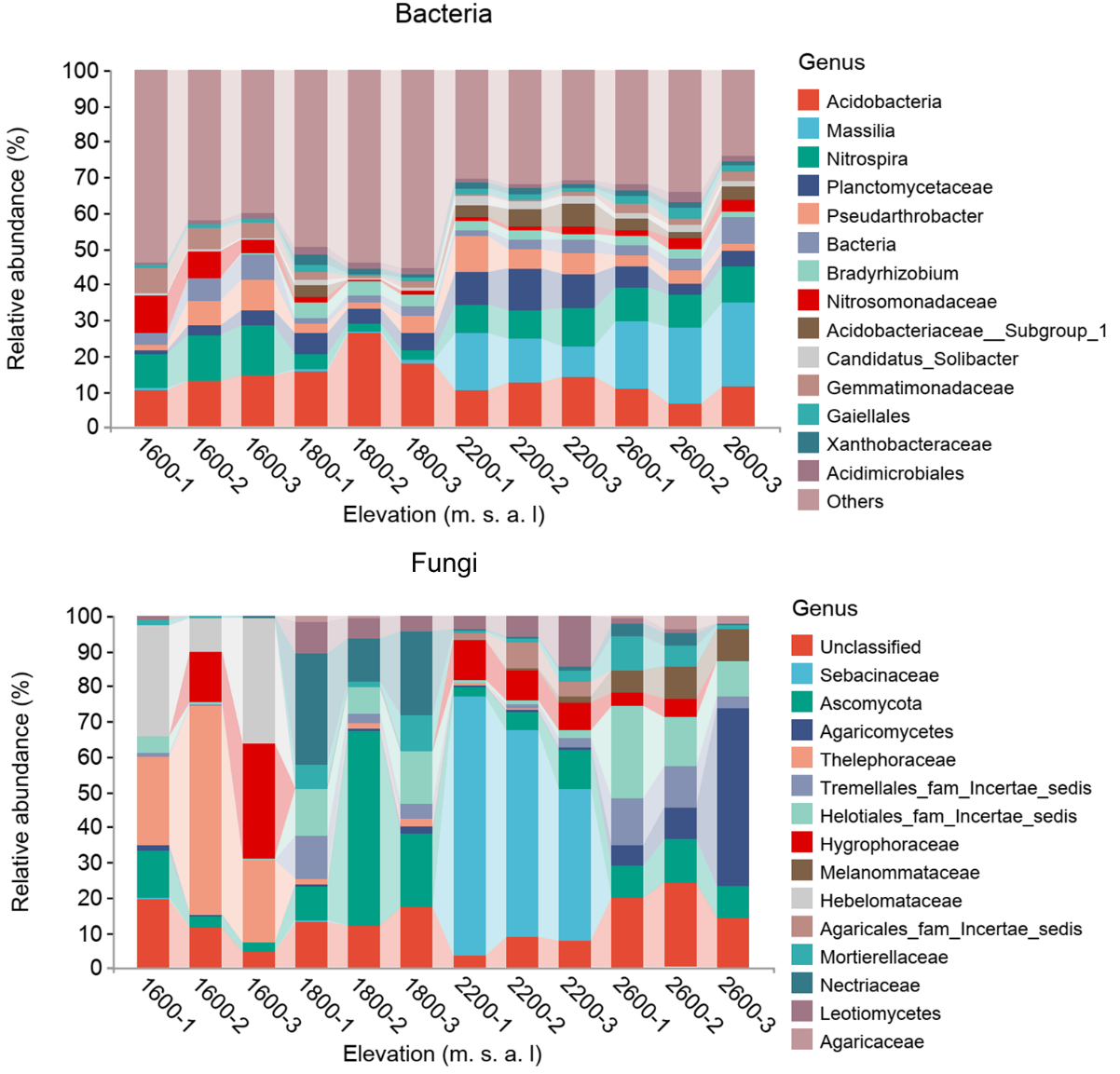


**Figure. S2** Soil bacterial and fungal community compositions at genus level along an elevation gradient in Snow Treasure Summit National Nature Reserve.

## Supplementary Tables

| Sites | Elevation (m) | Longitude | Latitude | Main vegetation | Soil type |
| --- | --- | --- | --- | --- | --- |
| 1600 | 1627.19 | 104°03'20" | 32°31'43" | *Alnus cremastogyne, Tetradium ruticarpum, Pteris multifida,* *Duchesnea indica, Boehmeria spicata, Artemisia carvifolia* | Mountain yellow-brown soil |
| 1800 | 1844.48 | 104°03'22" | 32°32'02" | *Magnolia officinalis, Avena fatua, Kolkwitzia amabilis, Anthriscus sylvestris, Cotoneaster horizontalis, Aralia chinensis, Cedrus deodara* | Mountain yellow-brown soil |
| 2200 | 2252.85 | 104°03'12" | 32°32'36" | *Phlomis umbrosa, Vitis amurensis, Spiraea salicifolia, Astilbe chinensis, Cyclosorus interruptus* | Mountain brown soil |
| 2600 | 2653.38 | 104°01'52" | 32°32'53" | *Cyclosorus interruptus, Rhododendron calophytum, Ligularia sibirica, Meconopsis chelidonifolia* | Mountain brown soil |

Table S1. Basic information of the sampling sites along mountain gradient.

1600: 1600 elevation, 1800: 1800 elevation, 2200: 2200 elevation, 2600: 2600 elevation.

Table S2. The Pearson correlation between soil enzyme activities and soil properties in Snow Treasure Summit National Nature Reserve.

|  | pH | SOC | TP | AP | TN | AN | TK | AK |
| --- | --- | --- | --- | --- | --- | --- | --- | --- |
| Sucrase | 0.43 | 0.091 | 0.527 | 0.367 | -0.01 | -0.062 | -0.051 | -0.024 |
| Catalase | 0.87** | -0.256 | 0.04 | 0.479 | -0.431 | -0.542 | -0.607* | -0.435 |
| β-glucosidase | 0.071 | 0.72** | 0.76** | 0.688* | 0.28 | 0.281 | -0.213 | 0.052 |
| Urease | -0.054 | 0.143 | 0.131 | -0.03 | 0.491 | 0.439 | 0.031 | 0.516 |
| ACP | -0.717** | 0.803** | -0.022 | 0.084 | 0.891** | 0.902** | -0.222 | 0.693* |

*, p<0.05; **, p<0.01. SOC: Soil organic carbon, TP: total phosphorus, AP: available phosphorus, TN: total nitrogen, AN: available nitrogen, TK: total potassium, AK: available potassium.

Table S3. The value of richness, evenness and Shannon diversity metrics among elevation gradient.

| Elevation | Bacteria | | | Fungus | | |
| --- | --- | --- | --- | --- | --- | --- |
|  | Chao1 | Shannon | Evenness | Chao1 | Shannon | Evenness |
| 1600-1 | 4653 | 6.00 | 0.710457 | 115 | 3.59 | 0.756597 |
| 1600-2 | 4932 | 6.82 | 0.802023 | 178 | 2.79 | 0.538425 |
| 1600-3 | 4125 | 6.76 | 0.812029 | 99 | 2.13 | 0.463535 |
| 1800-1 | 4264 | 6.83 | 0.817185 | 935 | 4.64 | 0.678308 |
| 1800-2 | 3707 | 6.64 | 0.807985 | 429 | 4.13 | 0.681354 |
| 1800-3 | 5112 | 6.89 | 0.806853 | 1068 | 4.71 | 0.67541 |
| 2200-1 | 4949 | 6.17 | 0.72529 | 572 | 2.46 | 0.387454 |
| 2200-2 | 5196 | 6.35 | 0.7422 | 600 | 3.22 | 0.503366 |
| 2200-3 | 3944 | 6.11 | 0.737927 | 588 | 3.20 | 0.501825 |
| 2600-1 | 5604 | 6.34 | 0.734541 | 550 | 4.01 | 0.635507 |
| 2600-2 | 6153 | 6.33 | 0.725527 | 630 | 4.22 | 0.654698 |
| 2600-3 | 3886 | 5.69 | 0.688434 | 353 | 2.85 | 0.485812 |

Table S4. Pearson correlation between microbial alpha diversity indices and environmental factors.

|  |  |  | pH | SOC | TP | AP | TN | AN | TK | AK |
| --- | --- | --- | --- | --- | --- | --- | --- | --- | --- | --- |
| Bacteria | Chao1 | r^2^ | -0.473 | 0.018 | -0.379 | -0.378 | 0.179 | 0.198 | 0.123 | 0.179 |
|  |  | *P* | 0.121 | 0.955 | 0.225 | 0.226 | 0.577 | 0.536 | 0.704 | 0.579 |
|  | Shannon | r^2^ | 0.474 | 0.219 | 0.148 | 0.642 | -0.381 | -0.415 | -0.670 | -0.605 |
|  |  | *P* | 0.119 | 0.494 | 0.646 | 0.024 | 0.221 | 0.180 | 0.017 | 0.037 |
|  | Evenness | r^2^ | 0.806 | 0.153 | 0.436 | 0.854 | -0.524 | -0.567 | -0.667 | -0.720 |
|  |  | *P* | 0.002 | 0.635 | 0.157 | 0.000 | 0.080 | 0.054 | 0.018 | 0.008 |
| Fungus | Chao1 | r^2^ | -0.528 | 0.820 | 0.354 | 0.275 | 0.475 | 0.518 | -0.086 | 0.200 |
|  |  | *P* | 0.078 | 0.001 | 0.259 | 0.387 | 0.118 | 0.084 | 0.790 | 0.533 |
|  | Shannon | r^2^ | -0.130 | 0.562 | 0.677 | 0.461 | -0.060 | 0.039 | 0.101 | -0.294 |
|  |  | *P* | 0.688 | 0.057 | 0.016 | 0.132 | 0.854 | 0.903 | 0.754 | 0.353 |
|  | Evenness | r^2^ | 0.310 | 0.193 | 0.737 | 0.510 | -0.424 | -0.343 | 0.133 | -0.554 |
|  |  | *P* | 0.326 | 0.548 | 0.006 | 0.091 | 0.169 | 0.275 | 0.681 | 0.061 |

Table S5. Pearson correlation between the relative abundance of microbial phyla and environmental factors.

|  |  | | pH | SOC | TP | AP | TN | AN | TK | AK |
| --- | --- | --- | --- | --- | --- | --- | --- | --- | --- | --- |
| Bacteria | Proteobacteria | r2 | -0.015 | -0.178 | 0.418 | -0.158 | -0.173 | -0.121 | 0.671 | -0.003 |
|  |  | *P* | 0.964 | 0.580 | 0.177 | 0.623 | 0.591 | 0.707 | 0.017 | 0.991 |
|  | Acidobacteria | r2 | -0.156 | 0.573 | 0.299 | 0.533 | -0.009 | 0.088 | -0.270 | -0.263 |
|  |  | *P* | 0.628 | 0.051 | 0.345 | 0.074 | 0.977 | 0.787 | 0.395 | 0.409 |
|  | Actinobacteria | r2 | 0.366 | 0.161 | 0.090 | 0.483 | -0.298 | -0.362 | -0.515 | -0.408 |
|  |  | *P* | 0.242 | 0.617 | 0.781 | 0.112 | 0.347 | 0.247 | 0.087 | 0.188 |
|  | Planctomycetes | r2 | -0.161 | 0.537 | -0.245 | 0.304 | 0.486 | 0.448 | -0.753 | 0.217 |
|  |  | *P* | 0.618 | 0.072 | 0.443 | 0.337 | 0.109 | 0.145 | 0.005 | 0.499 |
|  | Verrucomicrobia | r2 | -0.770 | 0.282 | -0.505 | -0.493 | 0.873 | 0.876 | -0.001 | 0.821 |
|  |  | *P* | 0.003 | 0.374 | 0.094 | 0.103 | 0.000 | 0.000 | 0.997 | 0.001 |
|  | Nitrospirae | r2 | 0.284 | -0.821 | -0.534 | -0.587 | -0.264 | -0.359 | 0.193 | 0.004 |
|  |  | *P* | 0.372 | 0.001 | 0.074 | 0.045 | 0.407 | 0.251 | 0.548 | 0.990 |
|  | Chloroflexi | r2 | 0.296 | -0.233 | -0.182 | 0.092 | -0.417 | -0.418 | -0.238 | -0.451 |
|  |  | *P* | 0.350 | 0.466 | 0.571 | 0.777 | 0.178 | 0.176 | 0.457 | 0.142 |
|  | Firmicutes | r2 | -0.271 | 0.010 | -0.257 | -0.335 | 0.484 | 0.441 | 0.046 | 0.546 |
|  |  | *P* | 0.394 | 0.975 | 0.421 | 0.288 | 0.111 | 0.152 | 0.888 | 0.067 |
|  | unclassified | r2 | 0.385 | -.682^*^ | -0.337 | -0.295 | -0.508 | -0.550 | 0.086 | -0.334 |
|  |  | *P* | 0.216 | 0.015 | 0.284 | 0.352 | 0.092 | 0.064 | 0.790 | 0.289 |
|  | Gemmatimonadetes | r2 | 0.889 | -0.524 | 0.111 | 0.234 | -0.645 | -0.751 | -0.214 | -0.552 |
|  |  | *P* | 0.000 | 0.081 | 0.732 | 0.465 | 0.023 | 0.005 | 0.503 | 0.063 |
| Fungus | Ascomycota | r2 | -0.083 | 0.588 | 0.692 | 0.588 | -0.113 | -0.006 | 0.033 | -0.335 |
|  |  | *P* | 0.798 | 0.044 | 0.013 | 0.045 | 0.727 | 0.984 | 0.919 | 0.287 |
|  | Basidiomycota | r2 | 0.121 | -0.499 | -0.682 | -0.468 | 0.134 | 0.019 | -0.178 | 0.315 |
|  |  | *P* | 0.708 | 0.098 | 0.015 | 0.125 | 0.678 | 0.953 | 0.581 | 0.318 |
|  | Funclassified | r2 | -0.078 | -0.508 | 0.067 | -0.528 | -0.289 | -0.225 | 0.791 | -0.070 |
|  |  | P | 0.810 | 0.092 | 0.835 | 0.077 | 0.363 | 0.481 | 0.002 | 0.829 |
|  | Zygomycota | r2 | -0.525 | 0.256 | 0.223 | -0.231 | 0.297 | 0.358 | 0.492 | 0.253 |
|  |  | *P* | 0.080 | 0.422 | 0.487 | 0.471 | 0.348 | 0.254 | 0.104 | 0.427 |
